# The Protective Effect of Nimodipine in Schwann Cells Is Related to the Upregulation of LMO4 and SERCA3 Accompanied by the Fine-Tuning of Intracellular Calcium Levels

**DOI:** 10.3390/ijms26020864

**Published:** 2025-01-20

**Authors:** Sandra Leisz, Saskia Fritzsche, Christian Strauss, Christian Scheller

**Affiliations:** Department of Neurosurgery, Medical Faculty, Martin Luther University Halle-Wittenberg, Ernst-Grube-Straße 40, 06120 Halle (Saale), Germany

**Keywords:** nimodipine, neuroprotection, cell death, Schwann cells, LMO4, SERCA, GSK3β, calcium signaling

## Abstract

Nimodipine is the current gold standard in the treatment of subarachnoid hemorrhage, as it is the only known calcium channel blocker that has been proven to improve neurological outcomes. In addition, nimodipine exhibits neuroprotective properties in vitro under various stress conditions. Furthermore, clinical studies have demonstrated a neuroprotective effect of nimodipine after vestibular schwannoma surgery. However, the molecular mode of action of nimodipine pre-treatment has not been well investigated. In the present study, using real-time cell death assays, we demonstrated that nimodipine not only reduces cell death induced by osmotic and oxidative stress but also protects cells directly at the time of stress induction in Schwann cells. Nimodipine counteracts stress-induced calcium overload and the overexpression of the Cav1.2 calcium channel. In addition, we found nimodipine-dependent upregulation of sarcoplasmic/endoplasmic reticulum calcium ATPase 3 (SERCA3) and LIM domain only 4 (LMO4) protein. Analysis of anti-apoptotic cell signaling showed an inhibition of the pro-apoptotic protein glycogen synthase kinase 3 beta (GSK3β). Nimodipine-treated Schwann cells exhibited higher levels of phosphorylated GSK3β at serine residue 9 during osmotic and oxidative stress. In conclusion, nimodipine prevents cell death by protecting cells from calcium overload by fine-tuning intracellular calcium signaling and gene expression.

## 1. Introduction

Nimodipine is a 1,4-dihydropyridine calcium channel antagonist blocking the influx of extracellular calcium through voltage-gated long-lasting (L)-type calcium channels. In this process, nimodipine binds to the α1 subunit, which consists of the transmembrane pore and voltage sensor of the calcium channel [1,2]. Four different L-type alpha1 subunits Cav1.1-Cav1.4 have been identified, which are differentially expressed in a tissue-specific manner [2]. Nimodipine mainly leads to the blockade of Cav1.2 and Cav1.3, which are predominantly found in the cell membrane of cardiovascular cells, neurons, and other cell types of the nervous system [3]. Due to its lipophilic molecular structure, nimodipine crosses the blood–brain barrier more effectively than other calcium channel blockers of the same class [4]. A clinical indication was found for nimodipine in the prevention of vasospasm and delayed cerebral ischemia neurologic deficit (DIND) in patients with aneurysmal subarachnoid hemorrhage (aSAH) [5,6,7], hypothesizing that nimodipine prevents the ischemic effects of vasospasm and DIND by relaxing cerebral vascular smooth muscle [8]. In addition, clinical trials have shown that prophylactic nimodipine treatment positively impacts nerve function preservation and regeneration in vestibular schwannoma (VS) and maxillofacial surgery [9,10,11]. Furthermore, a preoperative initiation of treatment demonstrated more beneficial results compared to intraoperative or postoperative administration. A pilot study, as well as a phase III multicenter clinical trial, indicated better hearing preservation with nimodipine after VS resection [12,13,14]. In previous in vitro studies, we demonstrated that nimodipine pre-treatment protects different cells of the central and peripheral nerve system during osmotic, oxidative, and heat stress [15,16]. Regarding the molecular mechanism of nimodipine treatment, the anti-apoptotic effect was accompanied by the activation of protein kinase B (AKT) and cyclic adenosine monophosphate response element-binding protein (CREB) signaling [16]. In addition, Koskimäki et al. demonstrated higher activation of tyrosine receptor kinase B (TrkB) receptor, AKT, and CREB [17]. In the present study, further mechanisms that may contribute to the neuroprotective properties of nimodipine were investigated. It was shown that nimodipine reverts the increased stress-induced intracellular calcium levels and regulates the expression of Cav1.2. Furthermore, a nimodipine-dependent upregulation of the LIM domain only 4 (LMO4) and sarcoplasmic/endoplasmic reticulum calcium adenosine triphosphatase 3 (SERCA3) protein, as well as an increased phosphorylation of glycogen synthase 3 beta (GSK3β) were detected.

## 2. Results

### 2.1. Nimodipine Pre-Treatment Protects the Cells at the Point of Stress Induction

**Figure 1:**
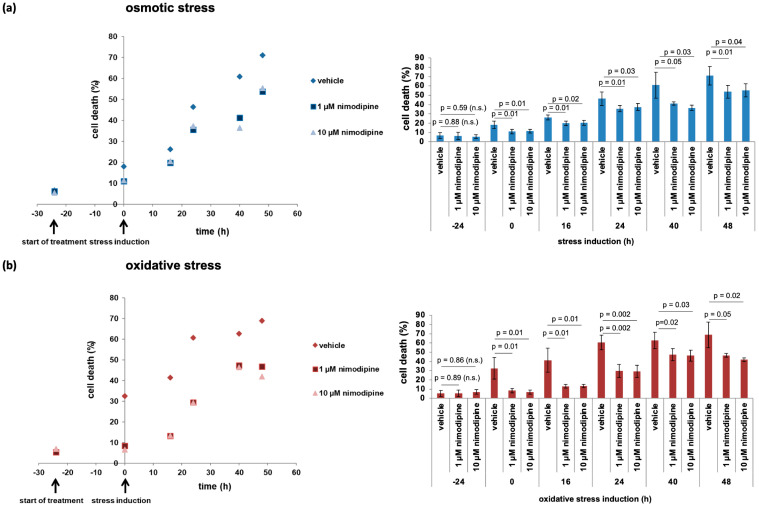
Real-time cell death analysis of nimodipine-treated Schwann cells during different stress conditions. SW10 cells were seeded in black flat 96-well plates and treated 24 h before stress induction with 1 µmol/L or 10 µmol/L nimodipine (start of treatment). Then, the cells were stressed with 150 mmol/L NaCl (osmotic stress) (**a**) or 2% EtOH (oxidative stress) (**b**) and were again treated with nimodipine. The cytotoxicity was measured by adding the DNA-binding fluorescence dye CellTox Green 1:1000 to the culture medium, and the fluorescence signal was measured at Ex/Em 485/535 nm. The fluorescence signal of the total lysed cells was set to 100%. The figure shows the means of three experiments with time (xy-plot) on the left side and a bar graph with standard deviations and statistical analysis on the right side. Statistical significance was assumed if the *p*-value was ≤0.05. n.s., not significant.

In recent years, nimodipine pre-treatment has been shown to reduce the death of nerve system cells during osmotic, oxidative, and heat stress [15,16]. The real-time cell death analysis offered the possibility to measure apoptosis from the start of treatment, 24 h prior to stress induction, up to 48 h after stress. At the beginning of treatment, the cell death rate of vehicle (6.63 ± 3.13%)- or nimodipine-treated Schwann cells (1 µmol/L, 6.24 ± 3.90%; 10 µmol/L, 5.64 ± 2.19%) did not differ (Figure 1). With osmotic stress induction, cytotoxicity was significantly reduced in the nimodipine-treated cells (1 µmol/L, 11.05 ± 2.52%; 10 µmol/L, 11.40 ± 2.08%; *p* = 0.01) compared to vehicle (18.10 ± 4.03%) (Figure 1a). Over time, the cell death rate of the vehicle-treated cells increased to 71.05 ± 10.06% after 48 h of stress incubation (16 h, 26.24 ± 2.69%; 24 h, 46.40 ± 7.25%; 40 h, 60.88 ± 13.92%), while the cells treated with nimodipine showed significantly reduced cell death at each measured time point. However, the level of dead cells increased under nimodipine treatment over the time of stress induction from 19.90 ± 2.42% (1 µmol/L) and 20.54 ± 2.67% (10 µmol/L) at 16 h of stress, to 35.61 ± 3.26% (1 µmol/L) and 37.41 ± 3.66% (10 µmol/L) at 24 h of stress, to 41.29 ± 1.68% (1 µmol/L) and 36.51 ± 2.73% (10 µmol/L) at 40 h of stress, and to 53.77 ± 6.73% (1 µmol/L) and 55.43 ± 7.16% (10 µmol/L) after 48 h of stress.

During oxidative stress induction (0 h, Figure 1b), the death of vehicle-treated cells increased to 32.50 ± 11.71% in comparison to nimodipine-treated cells (1 µmol/L, 8.45 ± 2.49%, *p* = 0.01; 10 µmol/L, 6.70 ± 2.30%, *p* = 0.01). Nimodipine treatment significantly decreased cell death at every observed time point. The cell death rate of the vehicle control increased from 41.43 ± 12.86% after 16 h to 60.59 ± 8.08% after 24 h, 62.37 ± 8.76% after 40 h, and 68.91 ± 13.83% after 48 h, whereas the cell death rate of the nimodipine-treated cells increased more slightly from 13.15 ± 1.88% (1 µmol/L) and 13.42 ± 1.91% (10 µmol/L) after 16 h, to 29.54 ± 7.08% (1 µmol/L) and 29.35 ± 6.53% (10 µmol/L) after 24 h, and to 46.71 ± 2.07% (1 µmol/L) and 41.94 ± 2.01% (10 µmol/L) after 48 h. Therefore, the highest impact of protective nimodipine treatment was observed at the stress induction time point during osmotic and oxidative stress.

### 2.2. Nimodipine Prevents Calcium Overload During Osmotic and Oxidative Stress

**Figure 2:**
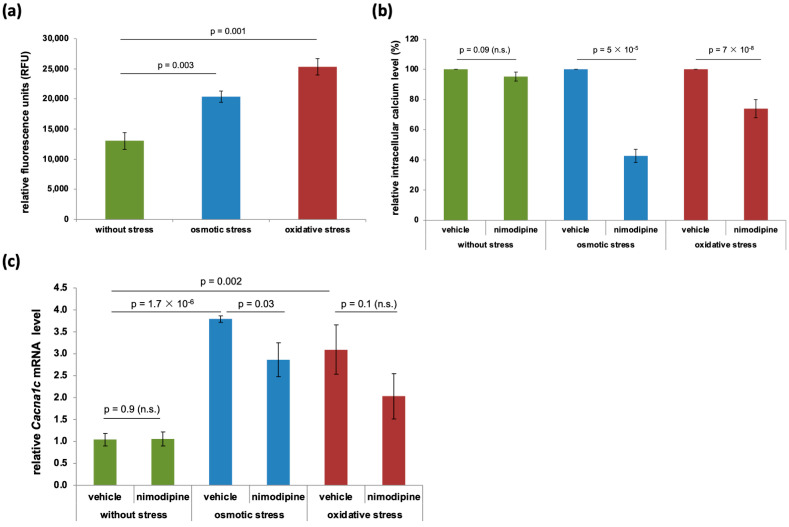
Intracellular calcium measurement during stress and nimodipine treatment. A total of 1 × 10^4^ SW10 cells were seeded in black flat 96-well plates. After 24 h, the cells were treated with 150 mmol/L NaCl (osmotic stress) or 2% EtOH (oxidative stress). A concentration of 10 µmol/L nimodipine was applied to the cells 24 h prior to stress and again at the time of stress induction. The intracellular calcium level was measured 24 h after stress induction via the incubation of cells with 5 µmol/L Cal-520 AM esters diluted in Hanks’ Balanced Salt Solution (HBSS). The fluorescence signal was detected at Ex/Em = 485/535 nm (**a**). The fluorescence signal of vehicle-treated cells was set to 100%, respectively (**b**). The mRNA level of *Cacna1c* was measured using real-time quantitative RT-PCR as described in the Materials and Methods Section (**c**). The graphs show the means and SDs of three experiments. Statistical analysis was performed with one-way ANOVA followed by Dunnett’s post hoc test. Significance was accepted if the *p*-value was ≤0.05. n.s., not significant.

Intracellular calcium overload is known to induce cell death [18,19]. The calcium channel inhibitor nimodipine can modulate calcium influx [20]. Intracellular calcium content was measured with Cal-520, a free-calcium-detecting fluorescence dye, as described in the Materials and Methods Section. The results showed an increased free intracellular calcium level of 36.01% (*p* = 0.003) from 13,046.67 ± 1411.97 relative fluorescence units (RFUs) to 20,388.33 ± 905.66 RFUs during osmotic stress and 49.52% (*p* = 0.001) to 25,338.33 ± 1387.11 RFUs during oxidative stress (Figure 2a). Unexpectedly, without cell stress, the nimodipine treatment (10 µmol/L) did not lead to a significantly reduced intracellular calcium concentration (Figure 2b). However, after 24 h of osmotic and oxidative stress, the nimodipine-treated cells showed a significant reduction in free intracellular calcium to 42.69 ± 4.46% (*p* = 5 × 10^−5^) and 74.00 ± 6.03% (*p* = 7 × 10^−8^).

Without stress, no regulation of the voltage-dependent L-type calcium channel subunit 1c (*Cacna1c*) mRNA levels by nimodipine was detectable. Osmotic and oxidative stress led to the higher expression of *Cacna1c*, which is the main protein of a nimodipine target channel (Figure 2c). Stress induction increased the relative *Cacna1c* mRNA level from 1.04 ± 0.14 (without stress) to 3.79 ± 0.07 (osmotic stress, *p* = 1.7 × 10^−6^) and 3.10 ± 0.56 (oxidative stress, *p* = 0.002). However, if Cav1.2 was blocked by nimodipine treatment (10 µmol/L), the *Cacna1c* mRNA level was decreased 1.5-fold (*p* = 0.03) during osmotic stress and oxidative stress (n.s., not significant) (Figure 2c). Voltage-dependent L-type calcium channel subunit 1d (*Cacna1d*), which is also blocked by nimodipine, was not expressed in the used cell models.

### 2.3. Analysis of Apoptosis- or Calcium-Signaling-Related Differentially Regulated Genes

During osmotic stress, we found a significant upregulation of brain-derived neurotrophic factor (*Bndf*, 1.6-fold), receptors for inositol 1,4,5-trisphosphate 2 (*Iptr2*, 4-fold), and *Cacna1c* (3.8-fold). A downregulation of *Lmo4* (1.7-fold), *activating* transcription factor 4 (*Atf4*, 1.7-fold), B-cell lymphoma-associated x (*Bax*, 1.5-fold), and B-cell lymphoma 2 (*Bcl2*, 2.5-fold) gene was shown through osmotic stress (Table 1). During oxidative stress, a significant upregulation of *Bndf* (1.7-fold), *Iptr1* (2-fold), *Iptr2* (3.6-fold), ATPase sarcoplasmic/endoplasmic reticulum calcium transporting (*Atp2a*) *2* (1.9-fold), *Atp2a3* (3-fold), *Cacna1c* (3.1-fold), DNA damage inducible transcript 3 (*Ddit3*, 3.3-fold), *Atf4* (1.7-fold), Bcl2-associated agonist of cell death (*Bad*, 2.3-fold), *Bax* (1.8-fold), and *Bcl2* (2.5-fold) was detectable (Table 1).

### 2.4. Nimodipine-Dependent Calcium Signaling Involved Differentially Regulated Genes and Proteins

**Figure 3:**
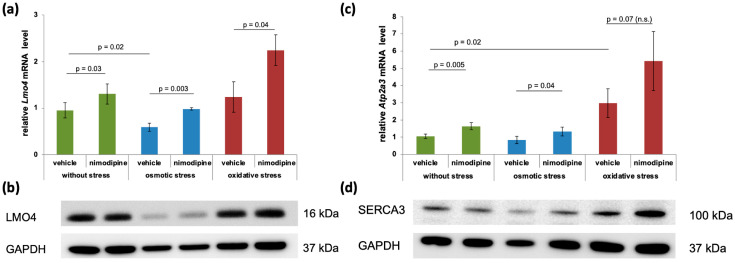
Nimodipine-dependent upregulation of intracellular calcium pump *Atp2a3* (SERCA3) and transcription co-activator LMO4. SW10 cells were treated stressed as shown in Figure S1. mRNA was isolated from the cells and transcribed into cDNA. *Atp2a3*, *Lmo4*, and *Gapdh* mRNA levels were detected via qPCR with target-specific primer (Table 1). The diagram shows the mean and standard deviations of three independent replicates. Significance was accepted if the *p*-Value was ≤0.05. n.s., not significant. (**a**,**c**). A total of 30 µg protein per lane was separated by SDS-PAGE and blotted onto a nitrocellulose membrane. The protein levels of LMO4, SERCA3, and GAPDH were detected via specific antibodies as described in the Materials and Methods Section (**b**,**d**). Antibody signals were detected via a CCD camera using the ECL method. The figure shows one representative immunoblot out of three independent experiments.

The results shown in Table 1 led to closer analyses of *Lmo4* and *Atp2a3* (Figure 3). The relative *Lmo4* mRNA level was weakly induced by nimodipine pre-treatment without stress (0.95 ± 0.16 to 1.30 ± 0.22, *p* = 0.03) and under osmotic stress (0.59 ± 0.09 to 0.98 ± 0.03, *p* = 0.003). The relative *Lmo4* mRNA level was about two-fold higher under oxidative stress (1.24 ± 0.32 to 2.25 ± 0.33, *p* = 0.04) (Figure 3a). Immunoblot analysis showed no visible induction of LMO4 protein levels without stress after nimodipine pre-treatment and under oxidative stress. Osmotic stress resulted in a significant reduction in LMO4. After nimodipine pre-treatment, an increase in the transcription co-factor was detectable for both osmotic and oxidative stress (Figure 3b).

Investigation of the relative *Atp2a3* mRNA levels showed an induction after nimodipine pre-treatment without cell stress (1.05 ± 0.13 to 1.64 ± 0.19, *p* = 0.005) and during stress conditions. Oxidative stress led to the highest increase in *Atp2a3* mRNA levels from 2.99 ± 0.83 to 5.43 ± 1.72 (n.s.), while cells stressed with 150 mmol/L NaCl showed an increase from 0.83 ± 0.21 to 1.33 ± 0.26 (*p* = 0.04, Figure 3c). On the protein level, the total amount of SERCA3 was not affected by nimodipine pre-treatment nor by the induction of oxidative stress. After osmotic stress, a reduction in SERCA3 was detectable, whereas pre-treatment with 10 µmol/L nimodipine led to an increase again. Similarly, SERCA3 protein levels increased after nimodipine pre-treatment in oxidatively stressed cells (Figure 3d). GAPDH served as a loading control.

### 2.5. Under Nimodipine, No Further Reduction in Intracellular Adenosine Triphosphate (ATP) Content Occurs, Already Triggered by Osmotic and Oxidative Stress

**Figure 4:**
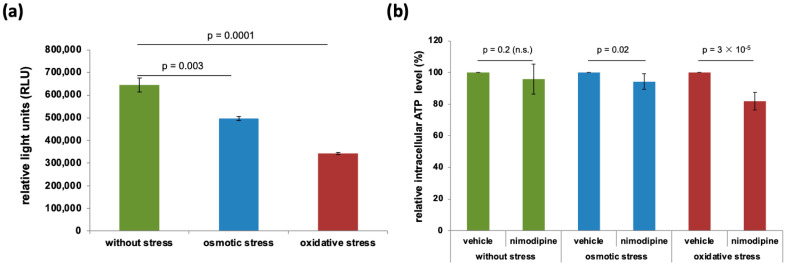
Intracellular ATP measurements during stress and nimodipine treatment. A total of 5 × 10^4^ SW10 cells per sample were seeded and treated as described in the Materials and Methods Section. The intracellular ATP content was measured using a CellTiter Glo luminescence assay. The plot of relative light units shows the influence of different stress conditions, which decreased under osmotic as well as under oxidative stress compared to untreated cells (**a**). To investigate the influence of nimodipine, untreated SW10 cells were set to 100% and compared to pre-treated cells, which showed no relevant decrease (**b**). The diagrams were obtained from three independent biological replicates. Statistical analysis was performed with one-way ANOVA followed by Dunnett’s post hoc test. Significance was accepted if the *p*-Value was ≤0.05. n.s., not significant.

To investigate the influence of the different stress conditions, the relative light units (RLUs) were compared to the measurement of ATP. A decrease in cellular ATP content was observed in SW10 cells without treatment (645,466.00 ± 30,047.49) compared to cells treated with 150 mmol/L NaCl (498,297.67 ± 8836.15, *p* = 0.003), and a higher reduction was detected under treatment with 2% EtOH (342,625.00 ± 4308.31, *p* = 0.0001) (Figure 4a).

To show the effect of nimodipine pre-treatment, the samples treated with and without stress (vehicle) were set to 100% and compared to the nimodipine pre-treated SW10 cells (Figure 4b). Without stress, there was no significant change in relative intracellular ATP levels after pre-treatment (95.82 ± 9.52%). In the cells stressed with 150 mmol/L NaCl, there was a slight reduction to 94.23 ± 4.83% (*p* = 0.03), and in EtOH-treated cells, the relative intracellular ATP level decreased to 81.92 ± 5.63% (*p* = 3 × 10^−5^) compared to the vehicle.

### 2.6. Nimodipine Pre-Treatment Led to an Inhibition of the Activity of GSK3β

**Figure 5:**
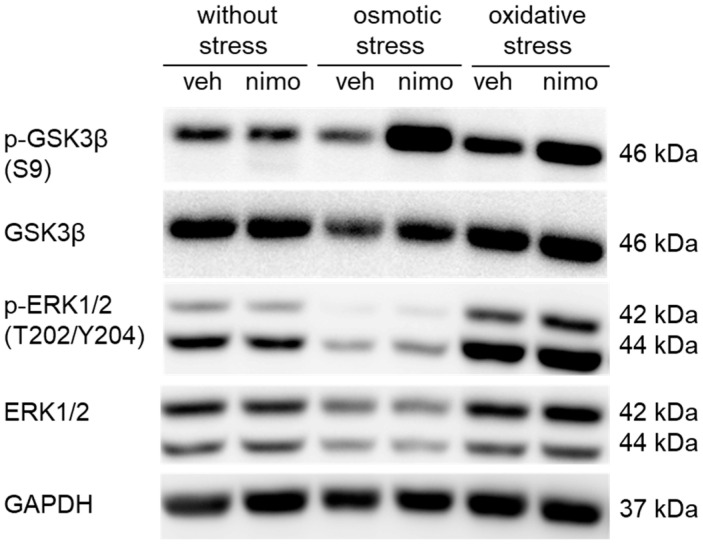
Nimodipine-dependent inhibition of GSK3β and ERK1/2 activity. A total of 30 µg per sample was loaded, separated by SDS-PAGE, and blotted onto a nitrocellulose membrane. GSK3β, ERK1/2, and their forms activated by phosphorylation p-GSK3β and p-ERK1/2 were detected with specific antibodies as described in the Materials and Methods Section. The presented illustration shows a representative example of three independent biological replicates.

Western blot analyses were used to investigate the influence of nimodipine pre-treatment under different stress conditions on GSK3β and ERK1/2 and their activation through phosphorylation (Figure 5). The phosphorylation of ERK at T202/Y204 leads to the activation of its kinase activity [21], whereas the phosphorylation of GSK at serine 9 causes an inhibition of its activity by blocking the substrate binding [22]. There was no change in the phosphorylation of GSK3β (S9) and ERK1/2 (T202/Y204) in SW10 cells pre-treated with nimodipine compared to those without pre-treatment (without stress). Exposure to osmotic stress resulted in a decrease in the activation of GSK3β and ERK1/2. However, treatment with nimodipine led to a significant increase in GSK3β activation and a slight increase in ERK1/2 activation. In EtOH-treated cells, higher phosphorylation of GSK3β and ERK1/2 was detected, which continued to increase with nimodipine pre-treatment compared to the control (without stress). The total amount of GSK3β and ERK1/2 remained constant without stress and under oxidative stress. Osmotic stress induced by 150 mmol/L NaCl showed a decrease in the total amount of ERK1/2, with nimodipine treatment leading to no change. The quantification of p-GSK3β and p-ERK1/2 is shown in Figure S2. GAPDH served as a loading control.

## 3. Discussion

Nimodipine is a calcium channel blocker with a good safety profile, which has been used in its clinical application for a long time and is therefore well characterized. The Food and Drug Administration (FDA) first approved nimodipine in 1988 [23]. Although its application has been primarily limited to SAH, nimodipine is also used for numerous off-label applications, such as assisting nerve preservation after vestibular schwannoma surgery [13]. By binding to the alpha subunit of the L-type calcium channel Cav1.2 and Cav1.3, nimodipine blocks Ca^2+^ influx into cells [1,2]. Interestingly, we observed a higher amount of free intracellular Ca^2+^ under stress conditions, which was reversed by nimodipine treatment. Furthermore, *Cacna1c* (Cav1.2) expression was induced by cell stress. However, nimodipine administration led to a reduction in stress-induced expression. Ca^2+^ is one of the most important intracellular messenger ions in the human body [18]. At the cellular level, Ca^2+^ can be derived from outside the cell through channels or released from internal stores via channels in the endoplasmic reticulum (ER) or sarcoplasmic reticulum (SR) [24,25]. The SR and ER are the main organelles for intracellular Ca^2+^ storage, with a physiological concentration of 1 mmol/L calcium [26]. Cell death or survival is regulated by intracellular Ca^2+^ homeostasis at the level of the SR/ER [27]. An increased intracellular calcium concentration has been shown to induce superoxide formation and trigger the release of pro-apoptotic proteins. It has also been associated with the decreased production of ATP and the activation of reactive oxygen species (ROS), which can trigger neuronal cell death [28]. Prolonged global increases in intracellular Ca^2+^ lead to autophagy, necrosis, and also programmed cell death, known as apoptosis [29]. Both this study and our previous studies have demonstrated that nimodipine exerts an anti-apoptotic effect under various stress conditions [15,16,30,31,32]. However, the precise underlying molecular mechanisms remain unclear. The real-time analysis conducted has demonstrated a preventive effect of nimodipine treatment. This suggests that, on the one hand, pre-treatment is essential for the protection of nerve tissue and, on the other hand, that nimodipine only exhibits its effect under stress induction. Under physiological conditions, the majority of free Ca^2+^ is stored intracellularly in the lumen of the ER. This Ca^2+^ is important for the cell to form a reservoir of signal ions and for protein synthesis and protein processing [33,34]. If the cytoplasmic Ca^2+^ concentration is too high, the mitochondria become overloaded, which results in the induction of apoptotic gene expression [19,35]. In detail, Ca^2+^ is released from the ER via the inositol 1,4,5-trisphosphate (IP3) receptor (IP3R) and transported to the mitochondria by the glucose-regulated protein 75 and the voltage-dependent anion channel in the mitochondrial membrane [36]. Ca^2+^ overload results in the opening of mitochondrial transition pores accompanied by the release of cytochrome c [37]. Thus, the release of Ca^2+^ from the ER is a crucial step in inducing the apoptosis cascade. We found a nimodipine-dependent upregulation of *Atp2a3* (SERCA3), *Iptr2* (IP3R2), *Bax*, and *Bcl2*. This indicates that nimodipine not only influences calcium transport via voltage-dependent calcium channels on the cell surface but also controls the intracellular release of calcium from the ER. Whether this is a downstream process of reduced calcium influx or if nimodipine directly binds to SERCA on the ER/SR membrane needs to be investigated in further experiments.

Regarding mitochondrial nimodipine-dependent regulated genes, members of the Bcl2 family of proteins are known to play an important role in modifying mitochondrial Ca^2+^ handling and restoring the Ca^2+^ balance [38]. The Bcl2 protein family is described as a rheostat, modulating pro-apoptotic and anti-apoptotic proteins [39]. Bcl2 inhibits apoptosis through interaction with IP3R, resulting in the inhibition of Ca^2+^ transfer to the mitochondria. Bcl2 also inhibits IP3R-induced autophagy [38]. Furthermore, Bcl2 has been shown to decrease Ca^2+^ efflux from the ER while maintaining ER Ca^2+^ storage [40], potentially by increasing the expression of SERCA [41] and/or preventing IP3R opening [42].

IP3Rs mediate these processes by controlling Ca^2+^ flux from the ER into the cytosol and mitochondria. Therefore, it is evident that survival- and death-promoting pathways and proteins can affect IP3R channels directly, which may occur in an IP3R isoform-dependent manner [27]. The regulation and activity of IP3R are controlled by IP3, cytosolic Ca^2+^, and the Ca^2+^ load in the lumen of the ER. Lower cytosolic Ca^2+^ leads to potentiation, while higher concentrations inhibit IP3-induced Ca^2+^ release from the ER. IP3R is mainly responsible for Ca^2+^ release from the ER. Ca^2+^ release from the ER via the IP3R is either induced by the binding of IP3 and/or Ca^2+^ itself, which further promotes Ca^2+^ release [43], although higher cytosolic Ca^2+^ concentrations can inhibit the IP3R [44].

Bad and Bax are known as pro-apoptotic members of the Bcl2 protein family [45]. The formation of a pore in the outer mitochondrial membrane, created by Bax and Bak, allows cytochrome c to be released into the cytoplasm, thereby activating the pro-apoptotic caspase cascade. Bad can form a heterodimer with Bcl2, which initiates Bax- and Bak-mediated apoptosis. However, phosphorylated Bad binds to protein 14-3-3, thereby exerting an anti-apoptotic effect [46]. Furthermore, Bak and Bax can translocate to the ER membrane and promote Ca^2+^ release from the ER lumen [47,48]. In turn, Ca^2+^ release from the ER recruits more Bax molecules from cytosol to the ER membrane [48]. However, the double knockout of Bax and Bak had reduced ER Ca^2+^ release and mitochondrial uptake, making the cells prone to apoptosis induced by ER Ca^2+^ release, and Bax targeting to mitochondria selectively restored apoptosis to BH3-only signals. Bax is described as a control point for ER Ca^2+^-dependent apoptosis [49]. These results indicate a need for further investigation into the influence of nimodipine on mitochondria-driven apoptosis. In particular, the phosphorylation of the Bcl2 protein family may provide insight into its activity.

Furthermore, ATP acts as a modulator of IP3R activity. Higher concentrations are inhibitory, while sub-millimolar concentrations increase IP3R activity [50,51]. IP3R2 exhibits the highest sensitivity with a 10-fold higher sensitivity to ATP than IP3R3 [52,53]. In our study, we found no induction of ATP levels during nimodipine pre-treatment. The intracellular ATP content was reduced during oxidative and osmotic stress, and it was also slightly reduced during nimodipine administration. This indicates that the cell-protective effect of nimodipine is not ATP-dependent. All three isoforms of IP3R have a phosphorylation site for AKT. Phosphorylation leads to AKT-promoted cell survival and is enhanced by Ca^2+^-induced conformational change in IP3R [54,55,56]. IP3R phosphorylation by AKT is stimulated in the presence of Ca^2+^. Triple IP3R knockout in pluripotent stem cells failed to generate Ca^2+^ signals and showed altered mitochondrial metabolism shifting to the pyruvate carboxylase pathway [57]. In our previous studies, we were able to show increased AKT activity by nimodipine under stress conditions [16,58]. The precise role of this mechanism and the function of intracellular calcium concentration in this process remain unclear. Further experimentation with AKT inhibitors, such as LY294002, and calcium agonists or *Cacna1c* knockout models may provide insight into these mechanisms.

SERCA is the only active Ca^2+^ transporter responsible for moving calcium from the cytosol into the SR/ER [26]. The expression of SERCA isoforms is tissue-specific [26]. SERCA3 has been identified in cell types such as lung cells, endothelial cells, and Purkinje neurons in the cerebellum [59]. Several studies have already explored the influence of SERCA expression on apoptosis. For example, increased SERCA2 expression in ovarian cancer cells has been shown to protect the cells from apoptosis [60]. On the other hand, the use of SERCA inhibitors such as casearin, thapsigargin, or kurahyne resulted in increased apoptosis in cancer cells accompanied by increased oxidative stress [61,62,63]. In another study by Liu et al., ER stress-mediated apoptosis was induced by ceramide in human adenoid cystic carcinoma cells via disruption of ER Ca^2+^ homeostasis, with a downregulation of mRNA expression of *Atp2a2* and *Atp2a3* [64]. On the other hand, triptolide was shown to induce apoptosis in PC12 cells through the upregulation of SERCA3 and an increase in Ca^2+^ [65]. These contradictory data suggest that the regulation of both IP3R and SERCA appears to be tissue-specific and isoform-dependent. In addition, cell signaling-related differences arise in the regulation of Ca^2+^ balance and fine-tuning of cancer cells compared with healthy tissue. Oxidative stress through the accumulation of ROS leads to SERCA reduction and dysfunction in hepatocytes [26]. It is thus necessary to ascertain whether nimodipine exerts a direct impact on the reduction in ROS and whether it possesses antioxidant properties.

In addition to proteins involved in the storage and release of calcium from the ER, we also investigated ER stress proteins. The data indicated an upregulation of *Ddit3* (protein name: C/EBP-homologous protein (CHOP)) and *Atf4* in response to nimodipine treatment. Both CHOP and ATF4 are induced by ER stress and are involved in regulating apoptosis [66,67]. Although CHOP, which is a target of *Atf4*, acts as a pro-apoptotic protein in various cell types [68,69,70], it appears to be protective in oligodendrocytes [71]. It is possible that CHOP target genes vary from cell to cell, suggesting that the targets of CHOP in oligodendrocytes could be different from those in other cell types. This supports the idea that CHOP induction in the context of unfolded protein response does not necessarily imply cell death [72]. The transcription factor ATF4 has also been shown to play a dual role in the modulation of cell death [67]. Otherwise, nimodipine treatment leads to ATF4 downregulation in the hippocampal CA1 area after chronic cerebral hyperperfusion in rats [73]. Therefore, it remains unclear whether nimodipine has a direct effect on *Atf4*.

LMO4 has a critical role in mediating the ototoxic and neurotoxic side effects of cisplatin [58,73]. The LMO4 knockout organ of Corti cells showed a decreased growth rate and migratory potential and enhanced susceptibility to cisplatin-induced cell death [73]. In a model of hyperphagia, LMO4-deficient paraventricular hypothalamus neurons showed reduced basal cellular excitability together with reduced voltage-activated Ca^2+^ currents. LMO4 regulates the expression of calcium channels involved in neuronal excitability [74]. LMO4 is required for insulin signaling in neurons and hepatocytes, which is accompanied by increased AKT serine 473 and GSK3β serine 9 phosphorylation [74]. In LMO4 knockout mice, the induction of the phosphoinositide 3-kinase (PI3K)/AKT signaling pathway is blocked by insulin. Since the effect of nimodipine pre-treatment appears to be similar to that of LMO4 at the molecular level, it may be likely that the upregulation of LMO4 plays a central role in the anti-apoptotic effect of nimodipine.

Nimodipine treatment attenuated chronic cerebral hypoperfusion-induced tau phosphorylation by an inhibition of GSK3β activation and neuronal apoptosis. These findings support a role for nimodipine inhibiting tau phosphorylation at Ser396 via miR-132/GSK-3β and may be a candidate for the treatment of tauopathy [75]. Our results also showed a nimodipine-dependent inhibition of GSK3β via enhanced phosphorylation at serine residue 9. This indicates that nimodipine could be used not only against nerve damage during surgical procedures, especially in neurosurgery, but also in the prevention of nerve damage in Alzheimer’s disease. In addition, there are some in vitro and animal studies suggesting the use of nimodipine in the treatment of multiple sclerosis [76,77,78]. Due to the lipophilic nature of nimodipine and its ability to cross the blood–brain barrier, the calcium channel inhibitor is suitable for use in both the peripheral and central nervous systems.

The study presented here confirms the neuroprotective effect of nimodipine under various stress conditions in vitro. The optimal concentration of stressors for investigating the mechanism has been identified in previous studies, yet it does not reflect the physiological conditions observed in nervous tissue. Subsequently, further in vivo studies are required to ascertain whether the observed effects and mechanisms can be verified.

**Figure 6:**
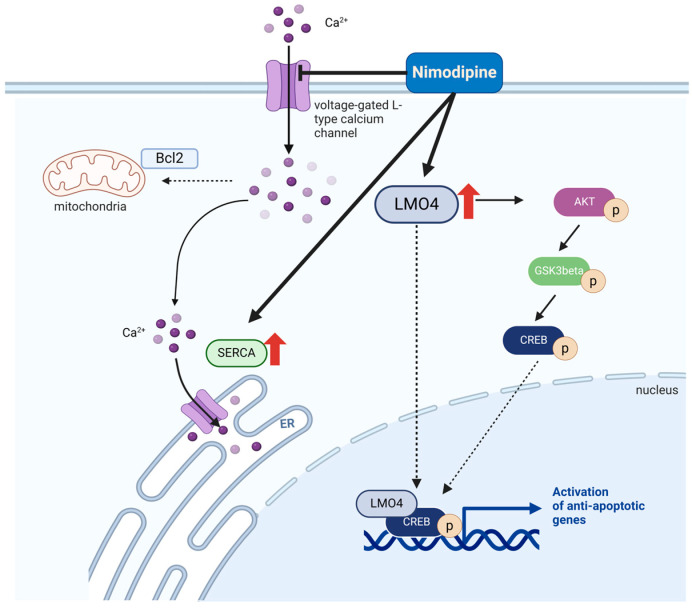
Scheme of nimodipine-dependent effects during cell stress. On the one hand, nimodipine blocks the increased calcium influx under various stress conditions. On the other hand, nimodipine pre-treatment leads to an enhanced expression of anti-apoptotic proteins such as LMO4 and SERCA3, the reduction in stress-induced calcium channel expression, and the increased activation of anti-apoptotic signaling pathways. Created with BioRender.com (agreement number: KG27BB04T2).

In summary, nimodipine was shown not only to activate the AKT and CREB signaling pathways but also induce the protein levels of LMO4 and SERCA3, whereby the increased expression of LMO4 could lead to the detected reduction in calcium channel expression and intracellular calcium levels under stress (Figure 6). There is also an increased inhibition of GSK3β, which represents a further mechanism in the reduction in cell death. However, whether LMO4 is functionally a key target protein in the molecular mechanism of action of nimodipine needs to be tested by the knockout and overexpression cell models.

## 4. Materials and Methods

### 4.1. Cell Lines

SW10 (Schwann cells) cells were cultured as recently described at 37 °C and 5% CO_2_ [16]. The experiments were carried out with at least three independent biological replicates.

### 4.2. Nimodipine Treatment and Stress Induction

SW10 cells were pre-treated with a vehicle (0.1% EtOH absolute) and 1 µmol/L or 10 µmol/L nimodipine (TCI Deutschland, Eschborn, Germany) for 24 h. Then, stress was induced with 2% (*v*/*v*) EtOH (oxidative stress) or 150 mmol/L NaCl (osmotic stress) by adding the substances to the cell medium. The experimental workflow is schematically shown in Figure S1.

### 4.3. Real-Time Cell Death Assay

Real-time cytotoxicity was measured with the DNA-binding dye CellTox Green (Promega, Mannheim, Germany) following the manufacturer’s instructions. In brief, 1 × 10^4^ SW10 cells per well were seeded in black 96-well plates (Greiner Bio-One, Kremsmünster, Austria) and treated with 1 or 10 µmol/L nimodipine. CellTox Green was diluted 1:1000 in cell culture medium without phenol red, and the fluorescence signal was detected at Ex/Em 485/535 nm with Tecan Reader F200Pro (Tecan, Crailsheim, Germany) 24 h prior to stress induction, directly after stress induction (0 h) and 16, 24, 40, and 48 h after stress induction. Real-time cytotoxicity was measured during oxidative and osmotic stress conditions. The background fluorescence of the culture medium was subtracted from each value, and the fluorescence signal of the total lysed cells was set to 100%.

### 4.4. RNA Isolation and Real-Time Quantitative RT-PCR

The total RNA of nimodipine and/or stress-treated SW10 cells was isolated with NucleoSpin RNA Plus Kit (Macherey & Nagel, Düren, Germany) following the manufacturer’s instructions. Then, 2 µg RNA was synthesized into full-length cDNA using the RevertAid First Strand cDNA Synthesis Kit (Thermo Fisher Scientific, Waltham, MA, USA) and oligo (dT)_18_ primer. The transcript levels were quantified with a specific primer (Table 2) and the Platinum™ SYBR™ Green qPCR SuperMix (Thermo Fisher Scientific, Waltham, MA, USA) using a two-step PCR program in the Rotor Gene Q cycler (Qiagen, Hilden, Germany). The annealing temperature of all used primers was 60 °C. GAPDH expression was used as a housekeeping gene. The relative values were calculated with three independent biological replicates using the ∆∆ct method.

### 4.5. Western Blot Analysis

Proteins were obtained as described [16], separated by SDS-PAGE, transferred onto a nitrocellulose membrane (Amersham, GE Healthcare, Freiburg, Germany), blocked for one hour in Tris-buffered saline (TBS) containing 0.1% (*v*/*v*) Tween^®^ 20 (Sigma Aldrich, Merck, Darmstadt, Germany) and 5% (*w*/*v*) skim milk (Carl Roth, Karlsruhe, Germany), and incubated at 4 °C with primary antibodies (diluted 1:1000 in blocking buffer) overnight. The next day, Western blots were washed five times with TBS containing 0.1% Tween^®^ 20 (TBS-T), incubated for one hour with secondary antibodies diluted 1:1000 in TBS-T supplemented with 2% skim milk, and washed again three times with TBS-T and at last two times with TBS. The specific antibody against SERCA3 was purchased from Thermo Fisher Scientific (Waltham, MA, USA), and the antibodies against phosho-GSK3β (S9, inactive state), total GSK3β, phospho-ERK1/2 (T202/Y204, active state), total ERK1/2, LMO4, GAPDH, and the Anti-IgG HRP-linked (mouse/rabbit) secondary antibodies were obtained from Cell Signaling Technology (Danvers, MA, USA). ECL signals were generated with Pierce ECL Western Blotting substrate (Thermo Fisher Scientific, Waltham, MA, USA) and measured via a CCD camera (ImageQuant LAS4000, GE Healthcare, Chicago, IL, USA). The quantification of blots was conducted with the software ImageQuantTL (version 10.2.499, Cytiva, Marlborough, MA, USA).

### 4.6. Intracellular Calcium Measurement

The calcium measurement was conducted in accordance with a previously described methodology [79]. Briefly, 1 × 10^4^ SW10 cells were seeded in black 96-well plates and treated with nimodipine and stress (Figure S1). Twenty-four hours after stress induction and nimodipine treatment, HBSS (Thermo Fisher Scientific, Waltham, MA, USA) without phenol red supplemented with 20 mmol/L 4-(2-hydroxyethyl)-1-piperazineethanesulfonic acid (HEPES, Lonza, Biozym, Hessisch Oldendorf, Germany), 0.02% Pluronic^®^ F-127 (Molecular Probes, Thermo Fisher Scientific, Waltham, MA, USA), 0.5 mmol/L probenecid (Sigma Aldrich, Merck, Darmstadt, Germany), and 5 µmol/L Cal-520 AM esters (Abcam, Cambridge, UK) was added to cells and incubated 90 min at 37 °C in the dark. Prior to fluorescence detection, the ester loading medium was replaced with HBSS containing 20 mmol/L HEPES and 0.5 mmol/L probenecid. Then, the fluorescence signal was measured at Ex/Em = 485/535 nm with Tecan Reader F200Pro. Wells with only medium served as the background signal.

### 4.7. ATP Measurement

The intracellular ATP content was measured using the CellTiter Glo luminescence assay (Promega, Mannheim, Germany). A total of 5 × 10^4^ SW10 cells were seeded in 24-well plates and treated as shown in Figure S1. Then, the medium was removed, and 200 µL of Cell Titer Glo substrate per well was added. Further, the plate was shaken orbitally for two minutes, and the lysed cell substrate mix was transferred into a white 96-well plate (Greiner Bio-One, Kremsmünster, Austria). The luminescence signal was detected with a microplate reader, Tecan F200Pro.

### 4.8. Statistical Analysis

Statistical analysis was performed via one-way ANOVA followed by Dunnett’s post hoc test (SPSS 25.0 Software, IBM, Ehningen, Germany). The vehicle-treated cells served as a control group. Significance was accepted if *p*-values were ≤0.05. The data were represented as the mean ± S.D.

## 5. Conclusions

Nimodipine has demonstrated neuroprotective potential in a variety of settings, including in vitro, in vivo, and clinical studies. To date, it is the only calcium channel inhibitor that has been employed in a clinical setting within the field of medicine pertaining to the nervous system. The present study shows that nimodipine not only regulates intracellular calcium concentration but also increases the gene expression of anti-apoptotic proteins such as SERCA3 and LMO4 and increases the activity of intracellular signaling pathways that reduce cell death. Moreover, nimodipine has been demonstrated to exert a cell-protective effect, necessitating pre-treatment. This provides new insight into the molecular mode of action of nimodipine and its neuroprotective effect and could provide an outlook for new clinical areas of application through further in vivo experiments in the future.

## Figures and Tables

**Table 1:** Differentially regulated nimodipine-dependent and stress-induced mRNA expression.

Functional Clustering	Gene Name (Protein)	Osmotic Stress		Oxidative Stress		Nimodipine Treatment					
						Without Stress		Osmotic Stress		Oxidative Stress	
		*fc*	*p*	*fc*	*p*	*fc*	*p*	*fc*	*p*	*fc*	*p*
Transcriptional regulator	*Lmo4*	**−1.7**	0.02 *	1.2	0.21	**1.4**	0.03 *	**1.7**	0.004 *	**1.8**	0.04 *
Neurotrophic factors	*Bndf*	**1.9**	0.01 *	**2.2**	0.003 *	1.4	0.25	1.2	0.33	1.2	0.16
	*Ntf4* (NT4)	1.2	0.68	2.3	0.06	1.2	0.20	−1.2	0.36	1.0	0.91
	*Ngf*	1.4	0.15	2.1	0.07	1.1	0.42	1.4	0.21	1.1	0.78
Calcium regulation	*Iptr1* (IP3R1)	−1.1	0.65	**2.0**	0.02 *	1.1	0.78	1.0	0.88	−1.3	0.32
	*Iptr2* (IP3R2)	**4.0**	0.04 *	**3.6**	0.002 *	1.9	0.15	**1.6**	0.04 *	1.3	0.20
	*Iptr3* (IP3R3)	−1.2	0.16	1.8	0.15	**1.3**	0.03 *	−1.1	0.52	−1.1	0.69
	*Atp2a1* (SERCA1)	1.3	0.60	1.8	0.37	−1.3	0.42	1.0	0.95	−1.1	0.85
	*Atp2a2* (SERCA2)	−1.1	0.25	**1.9**	0.03 *	1.1	0.08	1.3	0.15	1.1	0.69
	*Atp2a3* (SERCA3)	1.3	0.18	**3.0**	0.007 *	**1.6**	0.005 *	**1.6**	0.04 *	1.8	0.07
	*Cacna1c* (Cav1.2)	**3.8**	1.7 × 10^−6^ *	**3.1**	0.002 *	1.0	0.90	**−1.5**	0.03 *	−1.5	0.12
ER stress	*Ddit3* (CHOP)	1.1	0.56	**3.3**	0.03 *	**1.6**	0.007 *	1.6	0.10	1.2	0.58
	*Eif2ak3* (PERK)	1.1	0.34	**1.8**	0.01 *	1.3	0.11	−1.1	0.63	−1.1	0.74
	*Atf4*	**−1.7**	0.03 *	**1.7**	0.008 *	**1.5**	0.02 *	**1.7**	0.005 *	1.0	0.96
Mitochondrial apoptosis	*Bad*	1.3	0.14	**2.5**	0.03 *	1.1	0.07	1.4	0.19	1.1	0.78
	*Bax*	**−1.5**	0.05 *	**1.8**	0.002 *	**1.5**	0.003 *	**1.4**	0.01 *	1.3	0.07
	*Bcl2*	**−2.5**	0.001 *	**2.5**	0.02 *	**1.2**	0.01 *	0.9	0.76	1.1	0.86

Statistically significant differential gene expression is marked in bold. *fc*, fold change; ER, endoplasmic reticulum; *p*, *p*-value; * *p* ≤ 0.05.

**Table 2:** Primer used for quantitative real-time PCR.

Gene Name (Protein)	Oligo Sequence 5′ to 3′ (Forward, Reverse)	Reference Sequence
*Lmo4*	GCACGTCCTGTTACACCAAG,	NM_010723.3
	AGGGCTGTGGGTCTATCATG	
*Ngf*	CATCCACCCACCCAGTCTTC,	NM_013609.3
	GTGTGAGTCGTGGTGCAGTA	
*Bdnf*	TACCTGGATGCCGCAAACAT,	NM_007540.4
	AGTTGGCCTTTGGATACCGG	
*Ntf4* (NT4)	GTCCCCTGCGTCAGTACTTC,	NM_198190.1
	CCTGGGAGTCTGCAGTCAAC	
*Bax*	CCAAGAAGCTGAGCGAGTGT,	NM_007527.3
	TTGGATCCAGACAAGCAGCC	
*Bad*	ACATTCATCAGCAGGGACGG,	NM_007522.3
	ACTCATCGCTCATCCTTCGG	
*Bcl2*	GGGGTCATGTGTGTGGAGAG,	NM_009741.5
	TCAAACAGAGGTCGCATGCT	
*Atp2a1* (SERCA1)	AGTTTGACGATCTGCCCCTG,	NM_007504.2
	CCACAGCAGTACCAGATCCC	
*Atp2a2* (SERCA2)	GCGGTCCAAGAGTCTCCTTC,	NM_009722.3
	GGGCATCCTCAGCAAAGACT	
*Atp2a3* (SERCA3)	TTTTGAGTCACGCTTCCCCA,	NM_001163336.1
	AGACATCTGAAGCACCACCC	
*Iptr1* (IP3R1)	TGCTGCAAGGGTGATCTACG,	NM_010585.5
	TAACTCTCAGGCCCGGTGTA	
*Iptr2* (IP3R2)	GTGAAGGTGAAGGACCCGAC,	NM_019923.4
	TGATCCAAGAAAGCCCAGCC	
*Iptr3* (IP3R3)	ATCGACACCTTTGCCGATCT,	NM_080553.3
	GGCCGGTGTAGTCTGTCTTG	
*Cacna1c* (Cav1.2)	TTTCCAGATGAGACCCGCAG,	NM_009781.4
	AGAGGCAGAGCGAAGGAAAC	
*Cacna1d* (Cav1.3)	CGTTGCAGGCCTAGATTCCA,	NM_028981.3
	AGGAGCTTCGGTGTAGGGAT	
*Ddit3* (CHOP)	AGGAGAACGAGCGGAAAGTG,	NM_007837.4
	TCCGGAGAGACAGACAGGAG	
*Eif2ak3* (PERK)	AGCTGTGCAGGAAGGAGAAC,	NM_010121.3
	GCTTGGTCCCTACTTGTCCC	
*Atf4*	GCCTGACTCTGCTGCTTACA,	NM_009716.3
	GGTCATAAGGTTTGGGCCGA	
*Gapdh*	GCACAGTCAAGGCCGAGAAT	NM_001289726.1
	GCCTTCTCCATGGTGGTGAA	

## Data Availability

The original contributions presented in this study are included in this article and Supplementary Materials; further inquiries can be directed to the corresponding author.

## Supplementary Materials

The following supporting information can be downloaded at https://www.mdpi.com/article/10.3390/ijms26020864/s1.
