# Supplementary material for: The Protective Effect of Nimodipine in Schwann Cells Is Related to the Upregulation of LMO4 and SERCA3 Accompanied by the Fine-Tuning of Intracellular Calcium Levels
Source: Int J Mol Sci. 2025 Jan 20;26(2):864. doi: 10.3390/ijms26020864 (PMC11765607; doi:10.3390/ijms26020864)
Supplement: Supplementary file 1 [file ijms-26-00864-s001.zip › ijms-3394352-supplementary.pdf]

Supplementary Materials

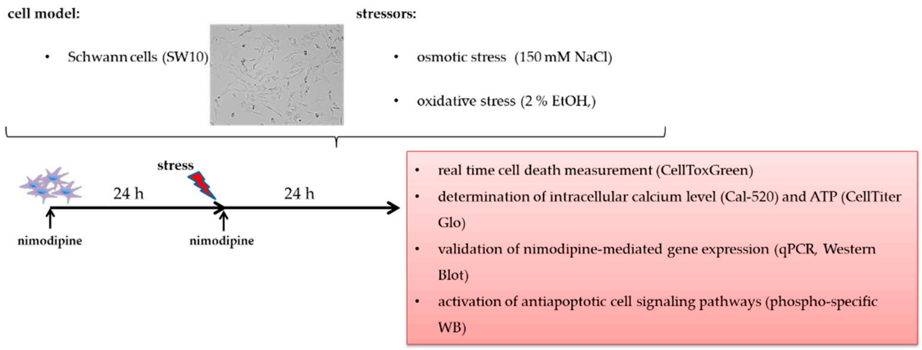

Figure S1. Schematic overview of methodical workflow of the study. Schwann cells were pretreated with different concentrations of the calcium canal antagonist nimodipine (10µM). After 24 hours stress was induced via 150 mM NaCl (osmotic stress) and 2 % EtOH (oxidative stress) while the same amount of nimodipine was added again. Another 24 hours later, real time cell death measurement as well as calcium assays (Cal-520) and ATP-assay, Western Blot and qPCR were performed. Three biological independent replicates were created for every described method.

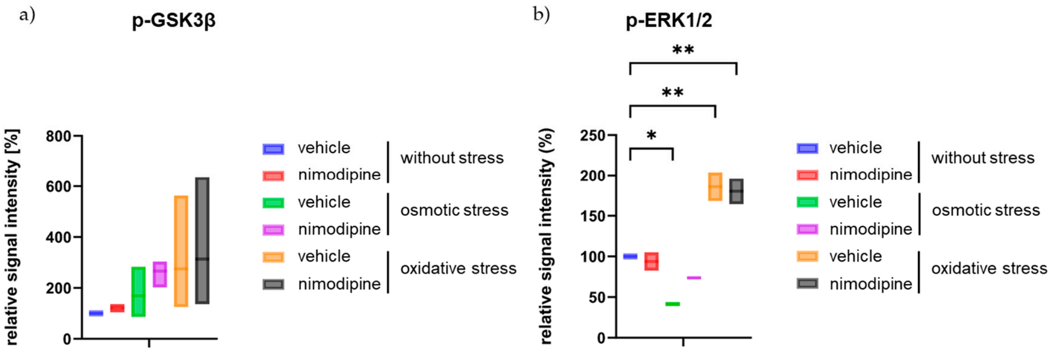

Figure S2. Quantification of phosphorylated protein levels of $GSK3\beta_{S9}$ (a) and $ERK_{T202/Y204}$ (b) after nimodipine pre-treatment and stress in Schwann cells. In order to determine the level of the protein that had undergone phosphorylation, the ratio of the phosphorylated protein to the total protein was calculated for each sample. The box plot shows the means and SD. * $p<0.05$, ** $p<0.01$
